# Where is the Planetary Boundary for freshwater being exceeded because of livestock farming?

**DOI:** 10.1016/j.scitotenv.2020.144035

**Published:** 2021-03-15

**Authors:** Guoyong Leng, Jim W. Hall

**Affiliations:** a Key Laboratory of Water Cycle and Related Land Surface Processes, Institute of Geographic Sciences and Natural Resources Research, Chinese Academy of Sciences, Beijing, China; b Environmental Change Institute, University of Oxford, Oxford OX1 3QY, UK

**Keywords:** Livestock, Water use, Feed crop, Global, Planetary boundary

## Abstract

Livestock production has significant impacts on the environment, including due to the use of water. In this study, we provide a spatially explicit estimation of livestock blue water use, by analyzing feed crop water use and livestock drinking water. For the past four decades, livestock water use has increased from 145 km^3^/year in 1971 to 270 km^3^/year in 2012 with an increasing trend of 1.36%/year. The proportion of livestock drinking water use has remained relatively stable at around 10% of total water use attributable to livestock production. Several hotspots of water use, including eastern China, northern India, US high plains, are identified in terms of the long-term averages, while South America and Central Africa show the most rapidly increasing trends. In USA, climate change is found to contribute most to the changes in water use attributable to livestock, while feed cropping intensity and land use change are the dominant driver in China and India, respectively. Though, in total, livestock water use makes a relatively modest contribution to the Planetary Boundary (PB) that has been proposed for anthropogenic water use (4000 km^3^/year), we argue that this aggregate number is not particularly meaningful, so we identify places where livestock is a major contributor to the unsustainable use of water, in northern India, part of the Middle East, Northern China and Central US. 7% of rivers where excessive water withdrawals mean that there is insufficient residual flow to sustain the aquatic environment (which we take to be the local manifestation of a PB) have been tipped over that boundary because of livestock farming, whilst in a further 34% of rivers, livestock farming on its own exceeds the water PB. Our results provide new and more geographically specific evidence about the impact that the meat industry makes on the PB for water.

## Highlights

• Global water use for feed crop production and livestock watering is mapped.
• The water issues associated with livestock production are spatially very variable.
• Livestock water use exceed the water Planetary Boundary in a third of farming areas.
• Using aggregate metrics of water footprint of meat and dairy products is misleading.

## Graphical abstract

**Figure:**
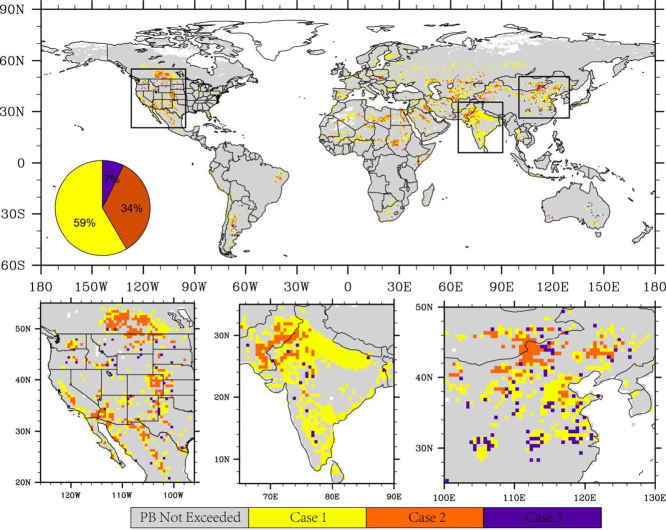

## 1 Introduction

Livestock production contributes 40% of value added by the agricultural sector and one-third of humanity's protein intake (Steinfeld et al., 2006), but at the expense of greenhouse gas emissions, pollution, and land conversion and degradation (Foley et al., 2011; Tilman et al., 2011). It is projected that global meat production could double by 2050s, and sustainable boundaries of the environment are expected to be exceeded (Pelletier and Tyedmers, 2010). Better understanding the environmental issues associated with livestock production is therefore important for defining the safe-operating planetary boundary (PB), which is fundamental for targeted adaptation and mitigation strategies towards sustainable development (Rockström et al., 2009; Steffen et al., 2015).

Among various environmental and health problems associated with livestock sector, freshwater use and related water problems have attracted increasing attentions (van Breugel et al., 2010; Springmann et al., 2018). It is recognized that animal products are very water-intensive (Mekonnen and Hoekstra, 2012), but little is known about the geographical patterns of livestock water use and the potential exceedance of water PB. Global hydrological models have been used as the major tools for estimating the geographical patterns of human water use worldwide (Alcamo et al., 2003; Pokhrel et al., 2012; Wada and Bierkens, 2014; Leng et al., 2015; Hanasaki et al., 2018). However, these have not focused specifically on the role of livestock, possibly because water withdrawals for livestock drinking water are relatively small. Previous global modeling efforts mainly focused on the simulation and analysis of irrigation water use and its impacts on the hydrological and climate systems (Fischer et al., 2007; Rost et al., 2008; Döll et al., 2012; Destouni et al., 2013; Leng et al., 2013; Leng et al., 2014; Jägermeyr et al., 2015).

Crops are used for various purposes including human consumed food, animal feed, biofuels, and other non-food products (Cassidy et al., 2013). Most of the blue water use along the supply chain of animal products goes to the growing of feed crops (Steinfeld et al., 2006). It is thus critical to differentiate feed crop water use from other purposes to enhance our understanding of water problems associated with the livestock sector. Addressing such issue is very important, given the fact that animal-food based diet has become increasingly poplar with growing populations and incomes, along with changing food preferences (Tilman et al., 2011). To date, however, globally explicit patterns of feed crop water use are under-examined along with its drivers and the impacts on water PB.

In this study, we fill the gap by providing a spatially explicit assessment of global livestock water use accounting for both livestock drinking water and feed crop water use, based on which the effects of livestock production in exceeding the water PB are evaluated. Here our focus is upon water that is withdrawn from freshwater sources – so-called ‘blue water’ (Mekonnen and Hoekstra, 2012) – for livestock production. We do not consider the rainwater that falls directly on pastures and arable feed crops – so-called ‘green water’ – because this water use has, at most, a marginal impact on freshwater scarcity. Specifically, we attempt to address three scientific questions; 1) how much water has been used for livestock production worldwide? 2) how different drivers have contributed to the recent changes in livestock water use? 3) how livestock water use has contributed to the exceedance of water PB? Through building a global modeling system, we identify the hotspots of livestock water use and the induced water problems. This paper is organized as follows: Section 2 introduces the dataset and methodology, Section 3 shows the results, Section 4 discusses the limitation and uncertainties of this study with conclusions made in Section 5.

## 2 Materials and methods

### 2.1 The Community Land Model

In this study, we use the Community land model version 5.0 (CLM) to simulate global irrigation water use and river flow (http://www.cesm.ucar.edu/models/cesm2/land/CLM50_Tech_Note.pdf). CLM is the land component of the Community Earth System Model (CESM) (Hurrell et al., 2013). It includes a crop model (Drewniak et al., 2013) coupled to the water, carbon and nitrogen dynamic processes, and allows for comprehensive examination of climate-water-crop interactions. At present, eight managed crop types (temperate soybean, tropical soybean, temperate corn, tropical corn, spring wheat, cotton, rice, and sugarcane) are explicitly represented within the model (Badger and Dirmeyer, 2015; Lawrence et al., 2019), with crop physiology and phenology algorithms derived from the AgroIBIS model (Kucharik and Brye, 2003). In CLM, irrigation is activated when crop leaf area index >0, and *β*_*t*_ < 1 (*i.e.* water stress limiting photosynthesis). Irrigation water demand depends on current soil water content and the target soil moisture, the latter of which is used for model calibrations (Leng et al., 2013; Leng et al., 2015). Comparing the simulated irrigation water use against census data from 346 administrative regions indicate that CLM after calibration can well reproduce the global patterns of irrigation water use (Leng et al., 2015). CLM also includes a river model called the Model for Scale Adaptive River Transport (MOSART) (Li et al., 2013; Li et al., 2015). Therefore, CLM allows for examination of water availibility constraint on irrigation water withdrawal in a coupled modeling system.

CLM solves land surface water and energy balances through a sub-grid hierarchy, and multiple plant functional types (PFTs) can exist in one soil column (Oleson et al., 2013). In the default settings, all crops are treated as unmanaged PFT. To activate the crop model, we partition the unmanaged crop PFT into various managed and unmanaged crop PFTs, with the fractions determined by the MIRCA2000 crop area map (Portmann et al., 2010). Specifically, the 5 arc minutes irrigated and non-irrigated crop areas are first resampled to the CLM grid, based on which the ratio of a specific crop area (*r*_*i*_) to the total area is calculated for each grid as:

(1) $$r_{i}=\frac{A_{i}}{\sum_{i}A_{i}}$$

where *A*_*i*_ is the crop area for crop type *i*. The ratios are used to partition the original unmanaged crop PFT (*PFT*_*generic*_*crop*_) to specific crop PFT (*PFT*_*crop*, *i*_) by:

(2) $${\mathrm{PFT}}_{\mathrm{crop},i}={\mathrm{PFT}}_{\mathrm{generic}\_\mathrm{crop}}\times r_{i}$$

The remaining crop area is treated as the unmanaged generic crop PFT (*PFT*_*generic*_*crop*_′) by:

(3) $${\mathrm{PFT}}_{\mathrm{generic}\_\mathrm{crop}}^{\prime }={\mathrm{PFT}}_{\mathrm{generic}\_\mathrm{crop}}-\sum_{i}{\mathrm{PFT}}_{\mathrm{crop},i}$$

The crop PFTs are further divided into irrigated and non-irrigated PFTs based on the ratios derived from Portmann et al. [2010] by:

(4) $${\mathrm{PFT}}_{\mathrm{irr},i}={\mathrm{PFT}}_{\mathrm{crop},i}*A_{\mathrm{irr},i}/A_{i}$$

(5) $${\mathrm{PFT}}_{\mathrm{noirr},i}={\mathrm{PFT}}_{\mathrm{crop},i}*A_{\mathrm{noirr},i}/A_{i}$$

where *A*_*noirr*, *i*_ and *A*_*irr*, *i*_ are the non-irrigated and irrigated areas for crop *i*, respectively. And the global distribution of gridded irrigated fraction is shown in Supplementary Fig. 1.

### 2.2 Gridded feed crop intensity and livestock intensity

At the field scale it is difficult to differentiate the crops that are used for feeding animals from others, as the ultimate destination of some crops is not necessarily known and there is not global tracking data at this scale. We therefore used the Food and Agriculture Organization's (FAO) Food Balance Sheets and trade statistics, which report crop production, exports, and domestic allocations at the national level. Following Cassidy et al. (2013), 41 major agricultural crops are analyzed, and the allocations of crop production to human consumed food, animal feed, biofuels, and other non-food products are quantified by:

(6) $$\mathrm{Crop}\;{\mathrm{allocation}}_{c,n}=\left[{\mathrm{production}}_{c,n}-{\mathrm{exports}}_{c,n}\right]\times \mathrm{domestic}\;{\mathrm{allocation}}_{c,n}+{\mathrm{exports}}_{c,n}\times \mathrm{importing nation}s^{\prime }\;a{\mathrm{llocations}}_{c}$$

where *Crop allocation*_*c*, *n*_ represents the crop uses *c* for a given nation *n*, and importing nations' allocations is a crop specific global average use of importing nations. Based on crop allocations, feed crop intensity is estimated as the relative proportion of crop production going to animal feed. The percentage fraction of crops used for feed is summarized in Supplementary Table 1. Since global census data is only available at the country scale, we are unable to quantify the within-country variability of crop allocations. Here, the country-level crop allocations are downscaled to the grid scale, assuming that grids within a specific country share the same allocation scheme.

We downscale the country-specific feed crop intensity to 0.5° using the spatial distribution of gridded crop areas from the MIRCA2000 map (Portmann et al., 2010). Country-scale livestock (cattle, buffalo, sheep, goats, pigs and poultry) intensity statistics obtained from FAOSTAT are downscaled to 0.5° according to the livestock density map (Robinson et al., 2014) as shown in Supplementary Fig. 1. We acknowledge that this approach of downscaling cannot reproduce the changes in the distribution within countries, but it reflects their large-scale temporal dynamics during the past decades.

### 2.3 Livestock water use and the drivers of its change

To estimate feed crop water use, we run the CLM for the period 1971–2012 at a spatial resolution of 0.5 degree to produce gridded estimates of irrigation water use. The CLM model is driven with meteorological forcing following the protocol of the Inter-Sectoral Impact Model Intercomparison Project (ISIMIP) (Warszawski et al., 2014), with initial conditions obtained from our previous studies (Leng et al., 2015). We limit our analysis to the year 2012 because the ISIMIP climate forcing data and other input parameters into our global model are available until 2012. Irrigation water use of the croplands is simulated under the fixed and transient scenarios. In the fixed scenario, land use patterns including irrigated area are kept constant and irrigation is driven only by climate variability. The transient scenario is the same as fixed scenario but using time-varying land use maps provided by the ISIMIP. The simulated irrigation water use is multiplied with feed crop intensity to estimate feed crop water use. Livestock drinking water amounts are estimated as a function of daily air temperature, following the approach by Steinfeld et al. (2006), which are combined with the corresponding numbers of livestock to estimate total livestock drinking water use. Though the temperature-based algorithm is widely adopted in global-scale modeling studies (Wada et al., 2014), we acknowledge that uncertainties would be introduced, as livestock drinking water requirement may be influenced by other factors besides temperature. However, we believe it would not affect our overall conclusions since the share of livestock drinking water use in total livestock water use is minor.

To understand the changes in feed crop water use, we develop a simple attribution method to quantify the contributions of changes in climate (CC), land use (LU) and feed crop intensity (FI) to the change rate of feed crop water use (FWU). This approach combines the variables of regional climate, land use and feed crop intensity in a causal relationship to feed crop water use. Based on our definitions, livestock water use can be regarded as the product of three variables:

(7) FWU=CC×LU×FI

The proportional change rate of a quantity *x*(*t*) is defined as *r*(*x*) = *x*^−1^*dx*/*dt*. From Eq. (7), the change rate of FWU can be estimated as:

(8) $$\left(\frac{\frac{\mathrm{dFWU}}{\mathrm{dt}}}{\mathrm{FWU}}\right)=\left(\frac{\frac{\mathrm{dCC}}{\mathrm{dt}}}{\mathrm{CC}}\right)+\left(\frac{\frac{\mathrm{dLU}}{\mathrm{dt}}}{\mathrm{LU}}\right)+\left(\frac{\frac{\mathrm{dFI}}{\mathrm{dt}}}{\mathrm{FI}}\right)$$

We then rewrite the proportional change rate of flood risk to:

(9) $$r\left(\mathrm{FWU}\right)=r\left(\mathrm{CC}\right)+r\left(\mathrm{LU}\right)+r\left(\mathrm{FI}\right)$$

Using annual time series of CC, LU and FI, we apply the above attribution method to quantify the contribution of each driver to the observed change rate of FWU. The theoretical proportional change rate represented by the sum of the proportional change rates of CC, LU and FI closely approximates the change rate of FWU. We first decompose the trend in FWU into three factors: CC, LU and FI, and then calculate their rates of change for a certain period using linear regression, which are normalized by their corresponding mean values. The relative contribution of each driver is estimated as the ratio of its proportional change rate to the proportional change rate of FWU during the same period.

### 2.4 Planetary boundary exceedance by livestock and non-livestock water use

We examine the role of livestock water use in exceeding of planetary boundary for water, and compare with that by the non-livestock water use. The planetary boundary for water has been challenged (Gleeson et al., 2020) because water is essentially a renewable resource so the impacts of human water use depend upon how much water is used, where and when. We therefore adopt a location-sensitive metric of sustainable water withdrawals. Following Gerten et al. (2013), we define the water PB on the basis of environmental flow requirement (EFR) for maintaining riverine ecosystems. The PB is considered to have been exceeded when anthropogenic water withdrawals are so great that the flow left in the river is less than the EFR. Here, we analyze three cases of the PB being locally exceeded: (1) PB exceedance (*i.e.* non-livestock+ livestock water use > PB) with non-livestock water use > PB and livestock water use < PB, which indicates the dominant role of non-livestock water use in leading to the exceedance of PB; (2) PB exceedance (*i.e.* non-livestock+ livestock water use > PB) with non-livestock water use > PB and livestock water use > PB, which indicates the equal role of non-livestock and livestock water use in leading to the exceedance of PB; (3) PB exceedance (*i.e.* non-livestock+ livestock water use > PB) with non-livestock water use < PB and non-livestock + livestock water use > PB, which implies that the PB would not have been exceeded without considering the role of livestock water use. We identify cases of non-exceedance of water PB (*i.e.* non-livestock + livestock water use < PB). The non-exceedance of PB indicates the safe space for water use, while case (1)–(3) demonstrate the relative roles of livestock and non-livestock in leading to the exceedance of PB. Water availability is estimated using the gridded annual runoff including surface flow and subsurface flow from the groundwater layer. Non-livestock water use is a combination of the non-feed crop water use simulated in this study and the industrial and domestic water use provided by the ISIMIP. The environmental flow requirement is estimated by multiplying the simulated water availability with the monthly fractions of environmental flow requirement produced by Pastor et al. (2014).

## 3 Results

### 3.1 Global livestock production and water use

**Fig. 1:**
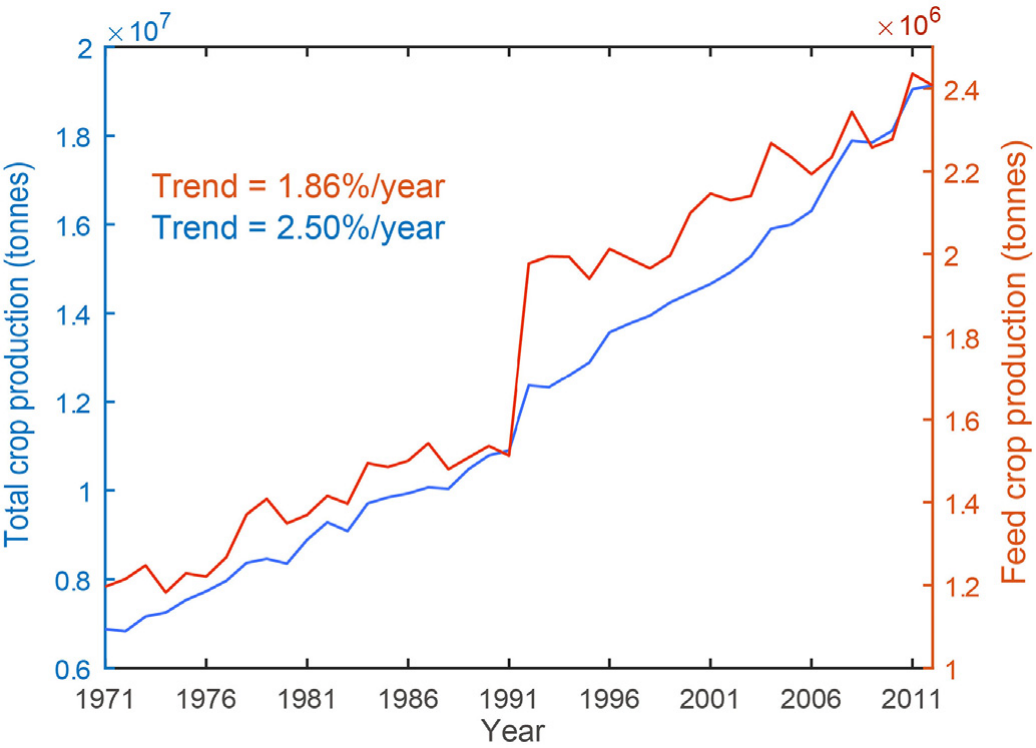
Temporal changes in global annual total production of all crops (blue line) and feed crops (red line) from FAO census data (http://www.fao.org/faostat/en/#data). (For interpretation of the references to colour in this figure legend, the reader is referred to the web version of this article.)

**Fig. 2:**
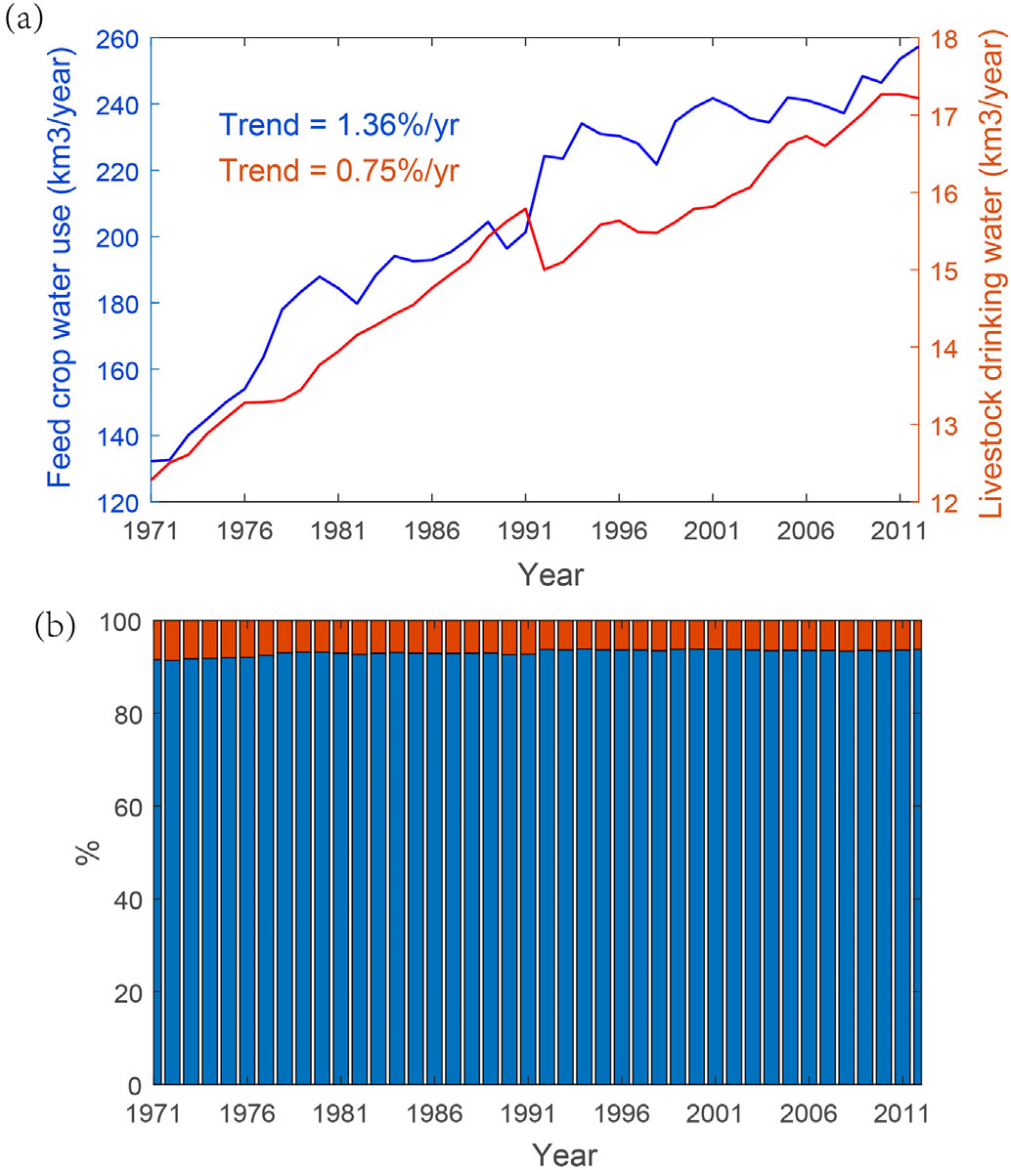
(a) Temporal changes in livestock drinking water (km^3^/year) and feed crop water use (km^3^/year), and (b) their relative shares (%) of the total livestock water use.

Fig. 1 shows the temporal changes in global annual total and feed crop production based on FAO census data. Though year-to-year fluctuations are observed, global crop production has increased at an average rate of 2.5%/year. Meanwhile, feed crop production has increased at an average rate of 1.86%/year, which is consistent with observed dietary changes shifting from grains to those that contain a greater proportion of meat, dairy, and eggs (Tilman and Clark, 2014; Godfray et al., 2018). By 2012, global total annual feed crop production amounted to 2.44 Billion tones, requiring an estimated 258 km^3^ water use (Fig. 2). According to our simulations, only 17.2km^3^/year of freshwater was used for the drinking purpose in 2012. During the past four decades, both feed crop water use and livestock drinking water have exhibited a significant increasing trend at a rate of 1.36%/year and 0.75%/year, respectively. However, the share of livestock drinking water to the total livestock water use remained relatively stable to be around 10%. Our estimates are broadly consistent with previous studies, demonstrating the relatively minor portion of livestock drinking water in livestock water use (Steinfeld et al., 2006). This suggests that previous estimations of water use of the livestock sector may have been largely underestimated, as only drinking water is explicitly accounted for in the livestock sector (Alcamo et al., 2003; Pokhrel et al., 2012; Wada and Bierkens, 2014; Leng et al., 2015; Hanasaki et al., 2018).

**Fig. 3:**
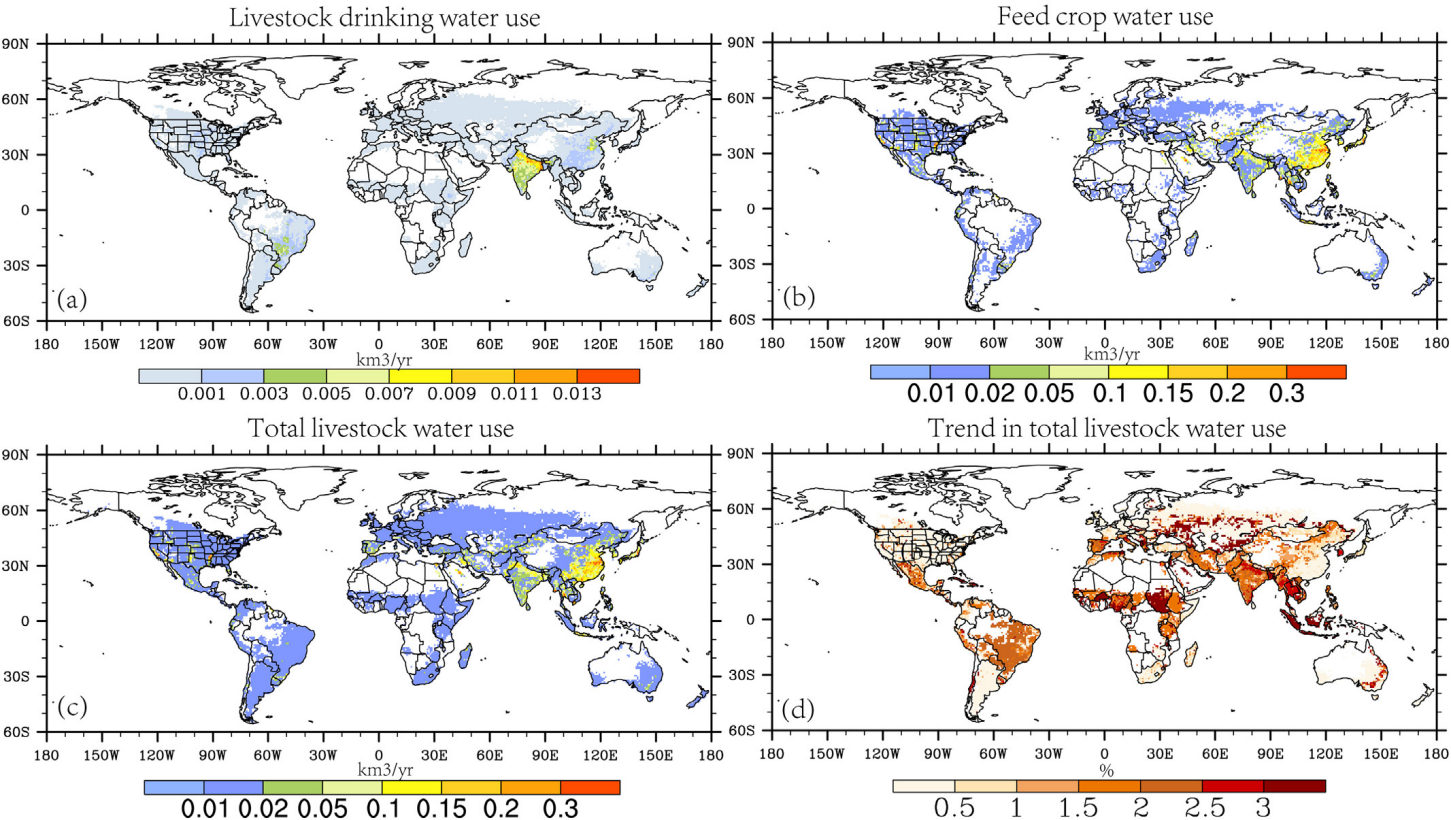
Spatial distribution of long-term mean (a) livestock drinking water (km^3^/yr), (b) feed crop water use (km^3^/yr), (c) total livestock water use (km^3^/year). (d) is the change rate (%) of total livestock water use for the period 1971–2012. Note the change trend is divided by its long-term average value of water use to estimate the relative change rate.

Previous studies have suggested that livestock water use mainly goes to feed crops, but it remains unclear about the geographical distribution pattern, and how it has changed in the past. Here, we have identified hotspots of livestock water use in eastern China, northern India, US central high plains (Fig. 3). India stands out as the top country in livestock drinking water, while China contributes the most of global feed crop water use. Overall, the spatial distribution pattern of total livestock water use is similar to that in feed crop water use, given its dominant role in contributing to total livestock water use. For the past four decades, an upward trend in livestock water use is detected for most of the feed cropping and livestock growing areas. Despite low estimates of long-term mean livestock water use, significant change trends are observed in most of Europe, Africa and South American countries. In parts of South Asia and Central Africa, livestock water use showed the largest increase rate up to 3%/year, with India identified as the hotspot not only in the long-term mean water use but also in its change rates.

**Fig. 4:**
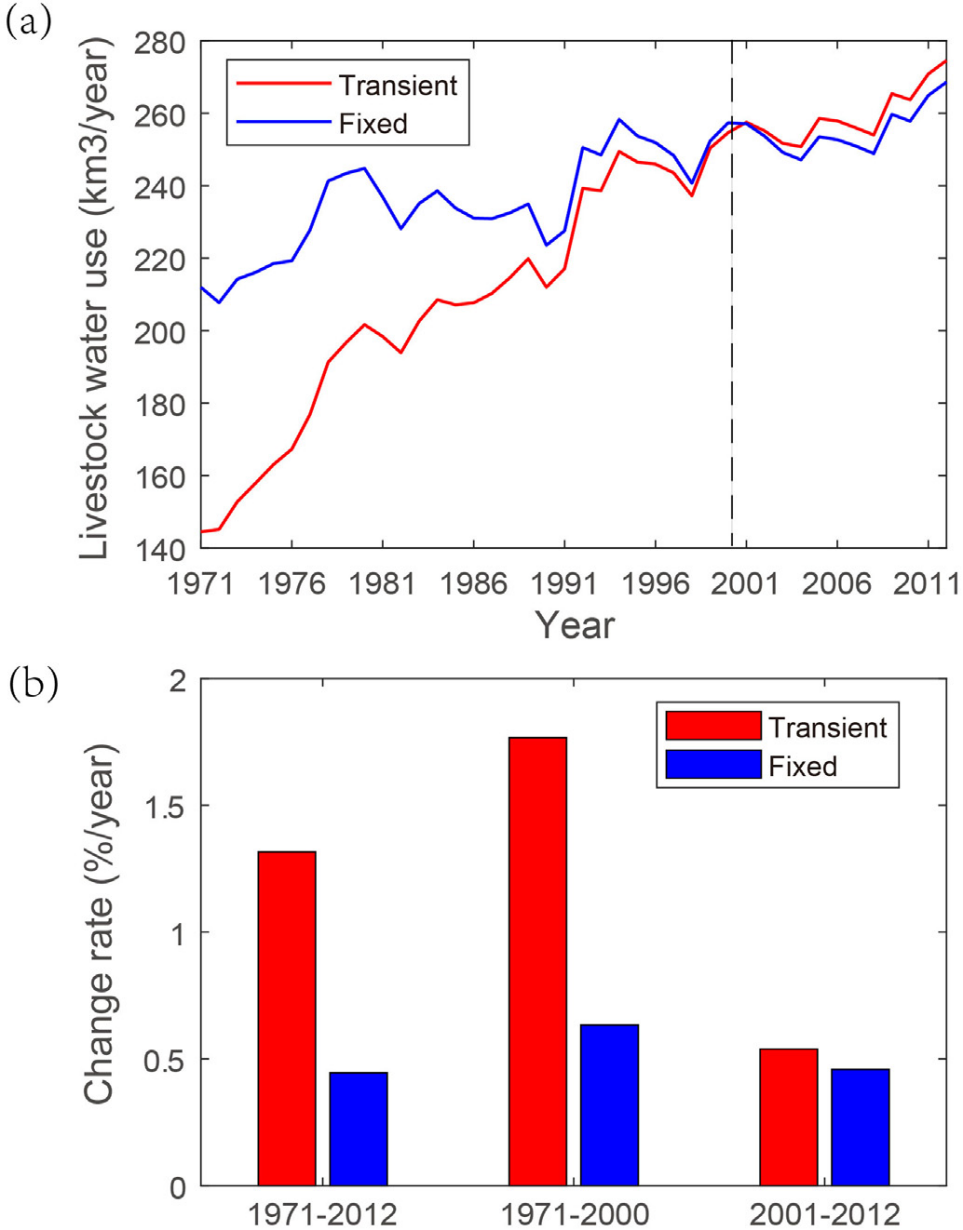
(a) Temporal changes in total livestock water use under present and transient social-economic conditions, and (b) the change rates for the whole period and two sub-periods.

For the globe as a whole, livestock water use has increased from 145 km^3^/year in 1971 to 270km^3^/year in 2012 with a change trend of 1.36%/year (Fig. 4), according to our simulations. Comparing the changes in livestock water use under the time-varying and fixed scenarios shows a more significant change trend under the scenario considering transient irrigated areas. This indicates that the historical increasing trend of livestock water use is mainly driven by the increase in irrigated areas. Specifically, when irrigation area is fixed around the year 2000, the simulated livestock water use exhibits a much smaller change trend at 0.73%/year, which is just half of the estimation under the transient conditions. In addition, comparing the estimations before 2000 and afterwards shows large differences in the detected change trends between the two sub-periods, with the change rate before the year 2000 tends to double that afterwards. This suggests that the overall increasing trend for the study period 1971–2012 is mainly contributed by the substantial increase in livestock water use during the period 1971–2000, which is associated with substantial expansion of irrigated area in developing countries.

### 3.2 Drivers of temporal changes in livestock water use

**Fig. 5:**
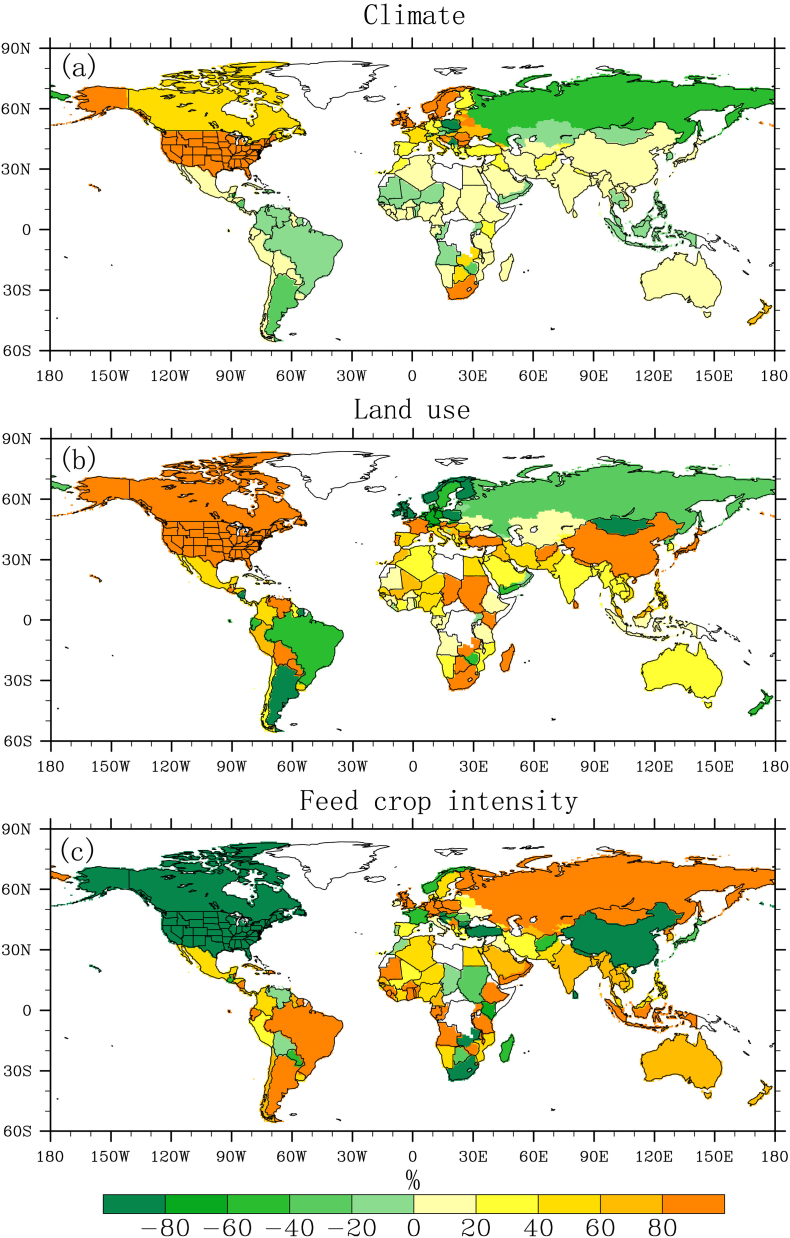
The relative contributions (%) of changes in (a) climate, (b) land uses and (c) feed crop intensity to the observed change rate of livestock water use for the period 1971–2012.

Fig. 5 shows the relative contributions of changes in climate, land use and feed crop intensity to the observed change rate of livestock water use for each country across the globe. It is found that climate change contributes the most to the changes in livestock water use in US, Northern Europe, with slight positive contribution observed in China, India and most of Africa (Fig. 5a). It is possible that recent climate change characterized with a warming climate has resulted in increase of evaporative demand of crops, so irrigation was used increasingly to minimize the negative climatic effects (Wada et al., 2013), thus leading to overall increase in feed crop water use. Land use change featured with a steady increase of irrigated areas (Supplementary Fig. S2) contributed positively to the changes in livestock water use in most of major livestock producing countries such as US, China and India (Fig. 5b). The negative impacts of climate and land use in South America and Northern Eurasian countries are due to the different sign of change trends between the two factors and livestock water use. Feed crop intensity has exerted a downward trend during the past decades (Supplementary Fig. S2), which led to an overall negative impact on livestock water use in US, Canada, China and parts of southern Africa, but with positive effects observed in other countries. Overall, the analysis suggests that recent changes in livestock water use are controlled by various factors, with distinct patterns in their relative contributions at the country scale.

### 3.3 The implications for water planetary boundary

**Fig. 6:**
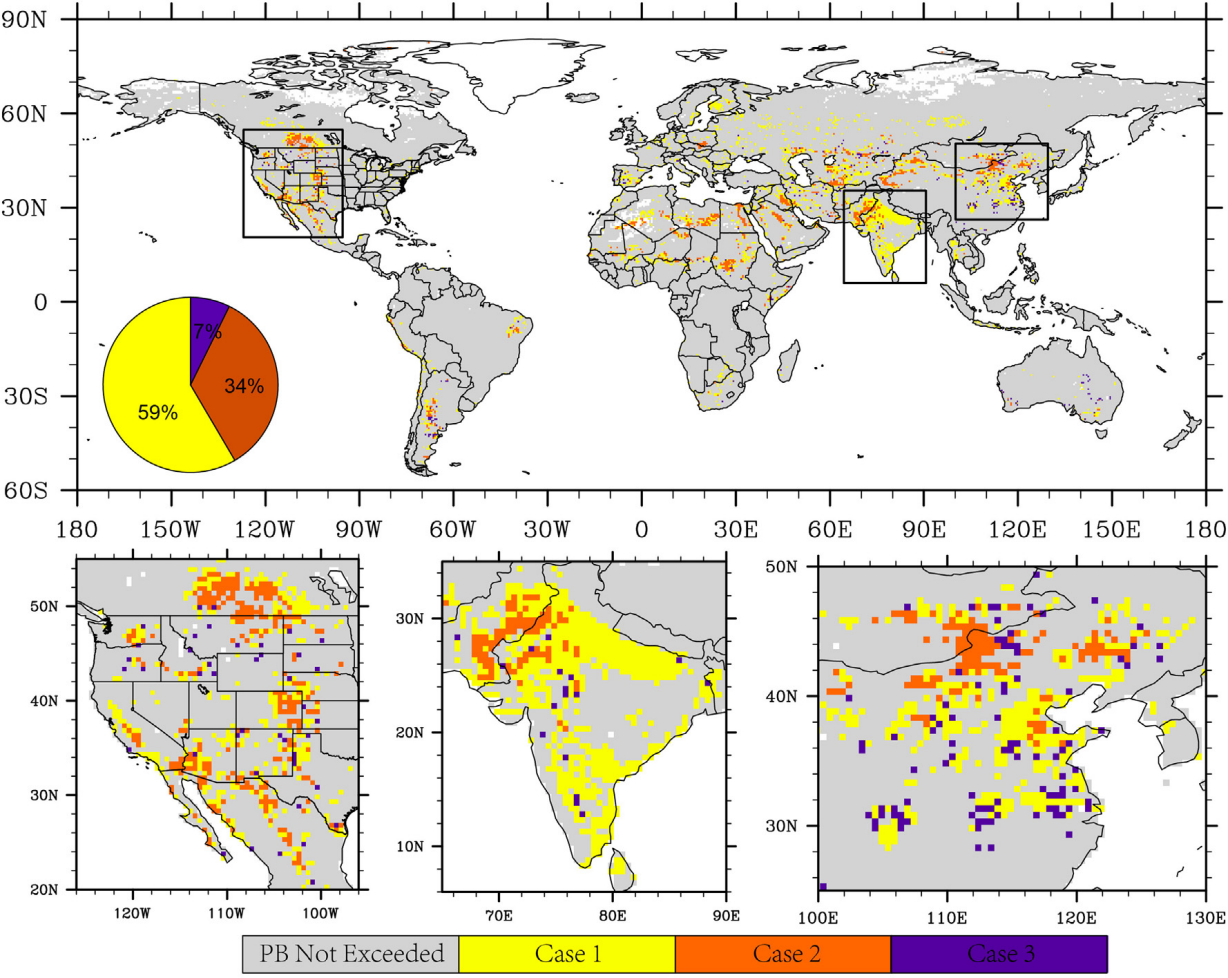
The state of water Planetary Boundaries for the globe and three hotspot regions. Grey: water PB not exceeded. Yellow: PB exceeded due to non-livestock water use (case 1). Red: PB exceeded both by non-livestock water use and by livestock water use (case 2). Purple: livestock water use has been responsible for tipping over the PB (case 3). Calculations are done for each year and the long-term mean is shown. The pie plot shows the percentage areas of each case. (For interpretation of the references to colour in this figure legend, the reader is referred to the web version of this article.)

As one of nine planetary boundaries, the water planetary boundary has attracted increasing attentions, but quantifications are mainly based on the aggregated value of total human water use (Rockström et al., 2009; Steffen et al., 2015; Springmann et al., 2018; Gerten et al., 2020; Gleeson et al., 2020; Zipper et al., 2020). Here, we identify where and how livestock water use has contributed to the exceedance of water PB, and compare with the role of non-livestock water use. It is shown that livestock water use has contributed substantially to the exceedance of water PB, though non-livestock water use dominates the global patterns (Fig. 6). In central High Plains, south America, northern India, livestock water use tends to exert comparable influence as non-livestock water use in leading to the exceedance of water PB. In summary, non-livestock water use alone has exerted the dominant influence in 68% of areas experiencing PB exceedance (case 1), while both livestock and non-livestock have exceeded the PB in 25% of the areas (case 2). Though relatively minor, the water PB would not have been exceeded without considering the contribution of livestock water use in the remaining 7% of the areas (case 3).

**Fig. 7:**
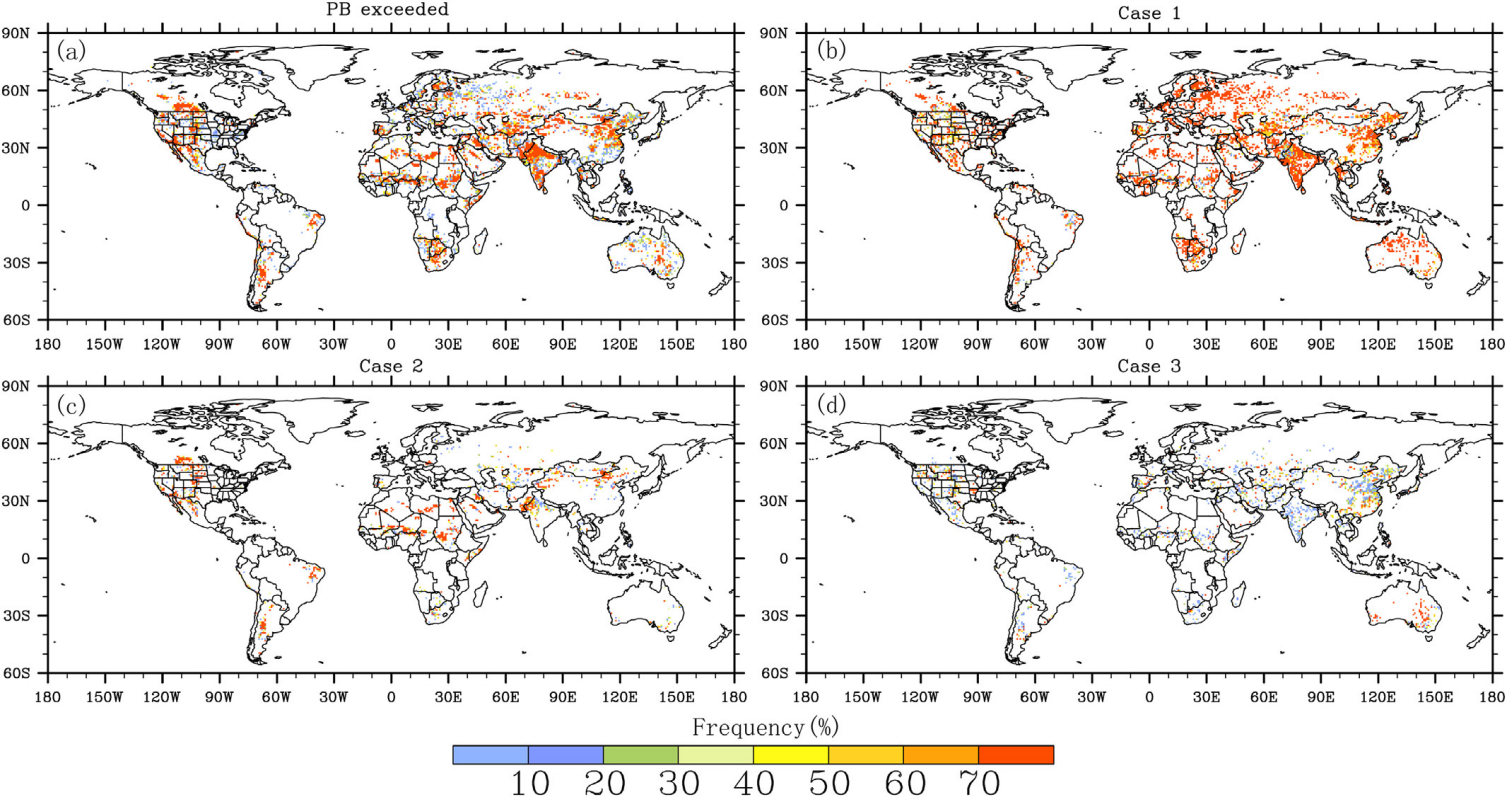
Global distribution of the annual frequency (%) of water PB exceedance. (a) shows the annual frequency of water PB exceedance relative to the whole study period, (b-d) divide the PB exceedance into three cases, and their frequencies relative to total water PB exceedances are calculated. Note that the occurrences of four cases are identified for each year, based on which the annual frequencies of each PB case are calculated for the study period.

Fig. 7 shows the status of water PB from the perspective of annual frequency. It is evident that more than 70% of years have experienced water PB exceedance during the past decades due to human water use (Fig. 7a), which is mainly contributed by non-livestock water use (*i.e.* case 1) (Fig. 7b). This is in line with the findings from the analysis of long-term mean PB status, as revealed in Fig. 6. However, some new hotspots tend to emerge when looking at the annual frequency of PB exceedance. Spatially, livestock water use has played an equally important role in contributing to the water PB exceedance in parts of US High Plains, Northern India, Northern China, Central Africa, South America, where the relative frequency of case 2 to total water PB exceedance is up to 70% (Fig. 7c). In areas including south China and Australia, about half of the water PB exceedance would not have been experienced without considering the role of livestock water use (*i.e.* case 3) (Fig. 7d). These new hotspot regions are, however, masked out when looking at the long-term mean picture (Fig. 6), pointing to the importance of time scale for examining the environmental issues induced by livestock water use. Overall, our results suggest that livestock water use has already contributed substantially to the water PB exceedance in several hotspot regions, which have great implications for enhancing our understandings of water PB and thus guiding sustainable development in the future.

## 4 Uncertainty and limitations

Livestock production impacts the aquatic environment in several ways, potentially contributing to water scarcity, pollution and sedimentation. This study represents the first global modeling estimation of livestock water use by explicitly considering both livestock drinking water and feed crop water use, through a synthesis of available census data and a process-based land model. However, several uncertainties have to be acknowledged when interpreting the results of this study.

First, our analysis of feed crop water uses and the related water issues relies on the process model used for the simulation of irrigation demand and water supply. The CLM model used in this study has been widely validated and is able to reproduce reported administrative-level irrigation water use (Leng et al., 2013; Leng et al., 2014; Leng et al., 2015), though local scale uncertainty is inevitable. Validation of river flow also indicates that the CLM performs well compared to observations (Li et al., 2015). Second, inherent uncertainty of grid-scale feed crop water use is expected, since feed crop intensity is estimated at the country-level without considering the spatial heterogeneity within the countries. To examine the uncertainty from this regard is difficult, as the only available world-wide data on crops used for various purposes is from the FAO country-level reports. Third, various assumptions of water supply have to be made when assessing the livestock induced water issues (Van Beek et al., 2011; Liu et al., 2017). On the one hand, neglecting non-renewable groundwater supply, water transfers between cells and rivers, and seasonality (Mekonnen and Hoekstra, 2016) would lead to overestimation of the water issues induced by livestock water use. On the other hand, assuming river water for the whole year can be stored for use would, in contrast, result in underestimation of water issues (Wada et al., 2011). These factors neglected in this study would therefore cancel out each other to certain extent, but require further investigations. Another important aspect of water supply uncertainty lies in the fact that water supply in reality is not only regulated by the natural water storage but also by the accessibility which is controlled by various social-economic factors (*e.g.* infrastructure, prices).

Therefore, we acknowledge that estimation of water PB exceedance induced by livestock water use should be improved in the future. Despite the above limitations, this study made a step forward by explicitly quantifying feed crop water use globally, which could greatly strengthen our understanding of livestock water use and enable the spatially explicit assessment of water PB in the livestock sector.

## 5 Summary and conclusion

Previous quantification of water PB is mainly based on global total human water use. However, unlike carbon emissions, it makes little sense to measure water use at an aggregate global scale – it needs to be assessed in relation to the amount of water that can be sustainable withdrawn from a given freshwater body. For the globe as a whole, the operating space for humanity remains safe, as the aggregate value of human freshwater use (2508 km^3^/year, of which 275 km^3^/yr is livestock water use) is lower than the threshold of 4000 km^3^/year proposed by (Rockström et al., 2009). However, we find that water withdrawals exceed sustainable levels in 8.65% of areas. In 59% of areas where the water PB has thus been exceeded this was not due to livestock (*i.e.* Case 1). Livestock water use alone is sufficient to exceed the PB in 34% of areas, though the PB is also exceeded by non-livestock water use in these locations (Case 2). In 7% of locations, livestock water use has been responsible for tipping over the PB (Case 3).

Despite several uncertainties, this study advances previous estimations of livestock water use by explicitly considering water use for feed crops, diagnosing the drivers behind its changes and evaluating its implications for water PB. Globally, livestock water use has exhibited a significant increasing trend at a rate of 1.36%/year, and the change rate before the year 2000 tends to double that afterwards. Our analysis demonstrates that the water issues associated with livestock production are spatially very variable. Several hotspots are identified including eastern China, northern India, US high plains from the perspective of long-term mean PB status. When looking at the annual frequency of PB exceedance, however, new hotspots including south China, Australia and Western US tend to emerge, where about half of water PB exceedance would not have been experienced without considering the role of livestock water use. These environmental impacts can be felt far from where animals are reared because feed crops, which account for about 90% of the water used in livestock production, are traded globally. Using aggregate metrics of the water footprint of meat and dairy products is misleading, because water use for livestock farming needs to be interpreted in the context of the amount of water that is available in different places, when other uses are also taken into account.

## CRediT authorship contribution statement

**Guoyong Leng**: Conceptualization, Investigation, Data collection, Methodology, Visualization, Writing - original draft, Writing - review & editing. **Jim W. Hall**: Supervision, Conceptualization, Investigation, Funding acquisition, Writing - original draft, Writing - review & editing

## Declaration of competing interest

The authors declare that they have no known competing financial interests or personal relationships that could have appeared to influence the work reported in this paper.

## Appendix A Supplementary information

Supplementary material Image 1
