# Supplementary material for: Where is the Planetary Boundary for freshwater being exceeded because of livestock farming?
Source: Sci Total Environ. 2021 Mar 15;760:144035. doi: 10.1016/j.scitotenv.2020.144035 (PMC8155394; doi:10.1016/j.scitotenv.2020.144035)
Supplement: Supplementary file 1 — Supplementary material [file mmc1.pdf]

Supporting Information for

Where is the Planetary Boundary for water being exceeded because of livestock farming?

Guoyong Leng[1,*] and Jim W. Hall[1]

[1]Environmental Change Institute, University of Oxford, Oxford OX1 3QY, UK

*Correspondence to Guoyong Leng (guoyong.leng@ouce.ox.ac.uk)

Contents of this file

Figures S1 to S2
Table S1

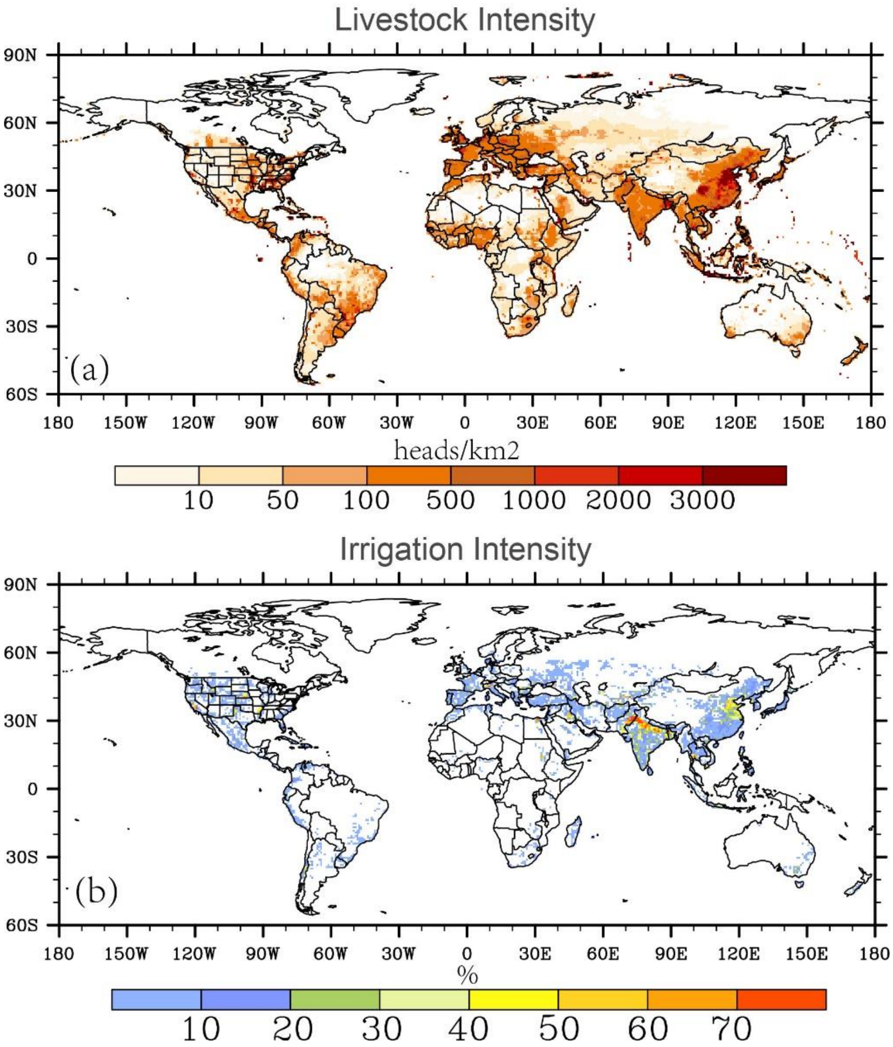

Figure S1 Global maps of (a) livestock intensity (heads/km2) from FAOSTAT and (b) irrigation intensity (%) from MIRCA2000.

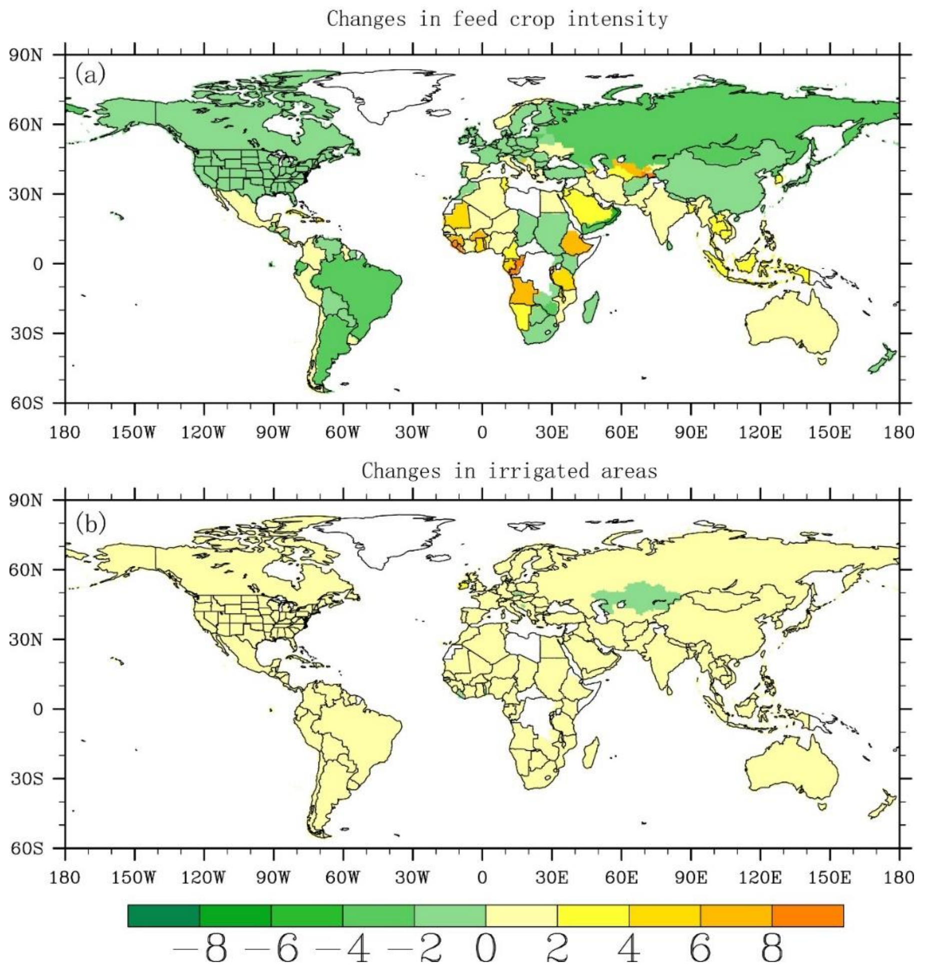

Figure S2 Change rates (%) in FAO-based country-level feed crop intensity and irrigated areas for the period 1970-2012.

Table S1 the crops analyzed in this study and the long-term mean global feed crop fractions (%).

Crop name	Feed crop fraction (%)
apples	0.90
bananas	1.30
barley	67.17
beans	5.86
cassava	33.21
cereals other	62.69
citrus other	0.00
coconuts	0.07
cottonseed	18.86
dates	4.32
fruits other	0.11
grapefruit	0.00
grapes	0.00
maize	64.09
millet	10.38
oats	78.06
oil crops other	7.63
olives	0.00
onions	0.09
oranges and mandarins	0.01
palm oil	0.00
peas	47.47
pineapples	0.00
plantains	12.36
potatoes	20.69
pulses other	20.82
rape and mustard seed	5.33
rice (paddy)	5.98
roots other	3.79

rye	45.86
sesame seed	0.01
sorghum	43.24
soybeans	3.94
sugar beet	2.85
sugar cane	2.19
sunflower seed	4.22
sweet potatoes	30.07
tomatoes	1.10
vegetables other	3.79
wheat	17.49
yams	13.03
